# Down-regulated lncRNA ROR in tumor-educated platelets as a liquid-biopsy biomarker for nasopharyngeal carcinoma

**DOI:** 10.1007/s00432-022-04350-1

**Published:** 2022-09-15

**Authors:** Jiazhou Wei, Xian Meng, Xiuqi Wei, Kaidong Zhu, Li Du, Hui Wang

**Affiliations:** 1 Department of Laboratory Medicine, Cancer Center, Union Hospital, Tongji Medical College, Huazhong University of Science and Technology, Wuhan, People’s Republic of China; 2 Department of Laboratory Medicine, Wuhan Jiangxia Hospital of Traditional Chinese Medicine, Wuhan, 430022 People’s Republic of China; 3 Division of Gastroenterology, Union Hospital, Tongji Medical College, Huazhong University of Science and Technology, Wuhan, 430022 People’s Republic of China; 4 Department of Laboratory Medicine, Union Hospital, Tongji Medical College, Huazhong University of Science and Technology, Wuhan, People’s Republic of China

**Keywords:** Tumor-educated platelets, lncRNA ROR, Liquid biopsy, Nasopharyngeal carcinoma, Diagnosis, Epstein–Barr virus

## Abstract

**Purposes:**

To evaluate the diagnostic value of tumor-educated platelets (TEP) lncRNA ROR for nasopharyngeal carcinoma (NPC).

**Methods:**

Quantitative real-time PCR was used to determine the expression level of TEP lncRNA ROR in NPC patients (*n* = 50) as compared to normal subjects (*n* = 33). The ROC curve analysis was performed to assess the diagnostic value of TEP lncRNA ROR for NPC. Correlations between TEP lncRNA ROR and clinical parameters were further analyzed.

**Results:**

The median of TEP lncRNA ROR was significantly lower in NPC patients than that in normal subjects (0.0209 vs 0.0610, *p* = 0.0019), while no significant difference was found in plasma lncRNA ROR. ROC analysis showed that TEP lncRNA ROR had a sensitivity of 60%, specificity of 70%, and accuracy of 63.9% in diagnosing NPC, and the area under ROC curve (AUC) was 0.70. The expression level of TEP lncRNA ROR in NPC showed no significant difference among different TNM stages. However, low level of TEP lncRNA ROR correlated well with positive Epstein–Barr virus (EBV) DNA (kappa value = 0.314, *p* = 0.06), TEP lncRNA ROR and EBV DNA had similar diagnostic positive rate (58.3%) for NPC, and the combination of TEP lncRNA ROR and EBV DNA increased the positive rate to 74%.

**Conclusion:**

The expression level of TEP lncRNA ROR was down-regulated in NPC and the diagnostic value of TEP lncRNA ROR was similar to EBV DNA. Our study indicated that TEP lncRNA ROR might serve as a novel type of liquid biopsy biomarker in diagnosis of NPC patients.

## Introduction

Nasopharyngeal carcinoma (NPC) is an epithelial carcinoma arising from the nasopharyngeal mucosal lining. NPC is characterized by a distinct geographical distribution and particularly prevalent in east and southeast Asia (Chen et al. 2019). The incidence of NPC is about 15 ~ 50/100,000 inhabitants in the southern region of China, Indonesia, Malaysia and South-East Asia (Shah et al. 2022). The tumor is often observed at the pharyngeal recess and have an insidious, asymptomatic development, resulting as either an incidental finding, or a late diagnosis of advanced and therapeutically unmanageable tumors (Moro et al. 2018). Early symptoms of NPC are not obvious, and patients at early stage can get a better prognose with the therapies like radiotherapy and chemotherapy. Therefore, early screen or diagnosis of NPC is of great significance for the prognosis of the disease.

Platelets are small cell fragments produced by megakaryocytes in the bone marrow. They play an important role in hemostasis and diverse thrombotic disorders (Zufferey et al. 2012). Although their central role is prevention of bleeding, platelets probably contribute to diverse processes in many diseases (Smyth et al. 2009). Platelets are anucleate cells, however, they contain a rich variety of RNAs and perform their own functions (Schedel Rolf 2009). During their lifespan, circulating platelets interact with immune cells, cancer cells, and stromal cells as well as communicate with vesicle in whole blood. These direct interactions and distant cell signaling change the content or function of platelets (Kuznetsov et al. 2012; McAllister, Weinberg 2014). Recent studies have found that tumor cells can influence platelets RNA content, resulting in tumor-mediated “education” of platelets. Alterations in the tumor-educated platelets (TEP) RNA profile have been described as a novel source of potential cancer biomarkers for early diagnosis and treatment monitoring (D’Ambrosi et al. 2021).

Long non-coding RNAs (lncRNAs) are non-protein coding RNAs with more than 200 nucleotides in length, and they are emerging key factors in the regulation of various cellular processes (Bergmann and Spector 2014). LncRNAs are involved in multiple diseases, such as heart diseases, genetic diseases and especially cancers (Batista and Chang 2013; Kazemzadeh et al. 2015). Accumulating evidences have shown that lncRNAs may play critical roles in multiple cancers and may offer new targets for their diagnosis, therapy and prognosis (Zhao et al. 2014). Long non-coding RNA regulator of reprogramming (lncRNA ROR) is a type of lncRNAs which has been identified as a promoter of human-induced pluripotent stem cells and participates in suppressing human embryonic stem cell self-renewal mediated by miRNA (Wang et al. 2013). Recent studies showed that upregulated lncRNA ROR in the liver tissue could serve as an unfavorable prognostic factor in various cancers (Lu et al. 2018). For example, Weiguo Wang found that lncRNA-ROR down-regulation inhibited osteosarcoma progression via YAP1 repression (Wang et al. 2021); Shuai Wang demonstrated that lncRNA ROR promoted multiple drug resistance (MDR) of gastric cancer cells and correlated with poor prognosis of patients with gastric cancer (Wang et al. 2020); Zheng found that lncRNA ROR regulated the proliferation and apoptosis of endometrial cancer cells via Notch1 protein (Zeng et al. 2020); Peng found that lncRNA ROR promoted estrogen-independent growth of breast cancer cells by regulating the ERK-specific phosphatase DUSP7 (Peng et al. 2017); Shen found that the level of plasma lncRNA ROR was upregulated in ovarian cancer and could be used as a potential biomarker for the diagnosis of ovarian cancer (Shen et al. 2020). Li found that lncRNA ROR expression increased in tumor tissues and can regulate the proliferation, migration and chemoresistance of NPC (Li et al. 2016). Hong found that lncRNA ROR could be down-regulated by polyphyllin I, and then suppressed tumor growth and induced apoptosis of NPC in vitro and in vivo through lncRNA ROR/P53 signal pathway (Hong et al. 2019).

These studies showed that lncRNA ROR was upregulated in tumor tissues and played an important role in the progression of NPC, however, whether the expression level of lncRNA ROR in TEP is altered remains unknown. In this study, we determined the expression level of TEP lncRNA ROR in NPC patients as compared with healthy controls and evaluated its diagnostic values. We found that TEP lncRNA ROR was significantly down-regulated in NPC, and showed similar diagnostic performance with Epstein–Barr virus (EBV) DNA, indicating that TEP lncRNA ROR might serve as a novel type of liquid biopsy biomarker for NPC diagnosis.

## Materials and methods

### Sample collection and platelet isolation

50 NPC patients and 33 healthy donors were included in the study. All samples were collected at Cancer Center, Union Hospital, Tongji Medical College, Huazhong University of Science and Technology. The study was approved by the Ethics Committee of Tongji Medical College, Huazhong University of Science and Technology (No: IORG0003571). Informed consent was obtained from all individual participants included in the study. The inclusion criteria of NPC subjects were as follows: 18–85 years old, newly diagnosed with a NPC, no radiotherapy or chemotherapy being received, and no other primary tumors.

2 mL of whole blood samples were obtained from NPC patients and healthy donors, and collected into test tubes containing EDTA-K_2_. The whole blood samples were centrifuged at 120 g for 20 min at room temperature, and got the supernatant containing platelet-rich plasma (PRP). Then, PRP was transferred to another centrifuge tube and centrifuged at 360*g* for 20 min at room temperature, getting the supernatant of plasma and precipitates of platelets. Next, 300 mL of plasma was pipetted from the supernatant and collected into a new centrifuge tube. Then, the platelets were washed twice with phosphate buffer saline (PBS). A high purity platelet was defined by less than 5 karyocytes per 107 platelets (Best et al. 2015). After centrifugation, platelets and plasma were added with 500 mL RNA lysate (a reagent that lysed cells and inactivated RNase included in a Liquid Total RNA Isolation Kit) and fully mixed for 5 min, respectively, then stored in − 80 °C refrigerator for later RNA extraction and amplification. The clinical and histopathological features of each participant, such as sex, age, tumor node metastasis (TNM) classification, history of EBV infection and pathological type, were recorded in detail.

### RNA extraction and relative real-time quantitative PCR (qPCR) analysis

Total RNA was extracted from isolated platelets or blood plasma using a Liquid Total RNA Isolation Kit (RP4002, BioTeke, Beijing, China). The quantity and quality of RNAs were determined by Nanodrop 2000 spectrophotometer and analyzed by an agarose gel electrophoresis. In total, 2 μg of RNAs were used for cDNA synthesis using a PrimeScript™RT reagent Kit with gDNA Eraser (RR047A, TAKARA, Dalian, China). Q-PCR was performed using iQ™ SYBR^®^ Green Supermix (BIO-RAD, CA, USA) on a BIO-RAD CFX96 system. H-actin was used as the internal amplification control. The primers used for amplification lncRNA ROR and H-actin are listed in Table 1. The relative expression levels of lncRNA ROR were calculated by 2^−△CT^ method; while △CT = CT _lncRNA ROR_–CT _U6_. EBV DNA levels were determined using EB viral nucleic acid quantitative fluorescent probe PCR assay (Shengxiang Biotechnology, Hunan, China) on the Stratagene Mx3000P system (Agilent Technologies, CA, USA) at Department of Laboratory Medicine of Wuhan Union Hospital.

**Table 1:** Primers used for cDNA synthesis and real time PCR

Primer	Sequence (5′ ~ 3′)
lncRNA ROR forward	CTCCAGCTATGCAGACCACTC
lncRNA ROR reverse	GTGACGCCTGACCTGTTGAC
H-ACTIN forward	AGCGAGCATCCCCCAAAGTT
H-ACTIN reverse	GGGCACGAAGGCTCATCATT

### Data analysis

SPSS21.0 software was used for statistical analysis. GraphPad Prism 7.0 software was used for receiver operating characteristic (ROC) curve analysis. Mann–Whitney *U* test was used to compare the expression level of lncRNA ROR in TEP or plasma between NPC patients and normal subjects. Kruskal–Wallis test, one way ANOVA or Mann–Whitney *U* test was used to compare the subtypes of TNM stages. The expression levels of lncRNA ROR were showed as [median, (lower quartiles, upper quartiles)]. Independent sample *t* test, Fisher’ exact test or Chi-square test for trend was used to determine the correlation between the expression level of lncRNA ROR and the clinical parameters of the subjects. The positive rates of lncRNA ROR and EBV DNA in the diagnosis of NPC were compared by McNemar and Kappa test. A two-sided test was performed in all statistical analyses. Statistically, significant difference was set at *p* < 0.05.

## Results

### Downregulation of TEP lncRNA ROR in NPC

**Fig. 1:**
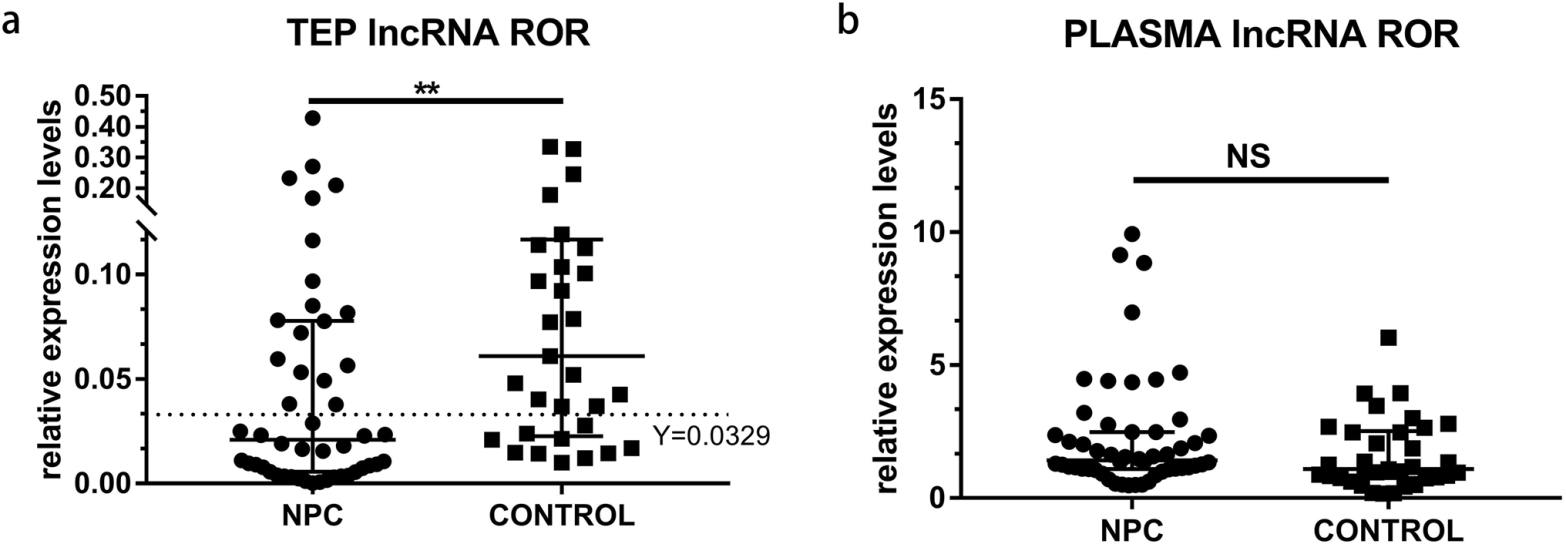
Relative expression level of lncRNA ROR in platelet and plasma of NPC patients and healthy donors. **a** The expression level of TEP lncRNA ROR in NPC patients were significantly lower than that in healthy donors. **b** There was no significant difference of plasma lncRNA ROR level between NPC and healthy donors. Mann–Whitney *U* test was used for analysis. **p < 0.01. *NS* no significance

The expression level of TEP lncRNA ROR in NPC patients was significantly lower than that in healthy controls [0.0209 (0.0056, 0.0777) vs 0.0610 (0.0226, 0.1166), *p* = 0.0019,] (Fig. 1a). However, there was no significant difference in the expression level of lncRNA ROR in plasma between NPC patients and normal subjects [1.4200 (1.0980, 2.4830) vs 1.1000 (0.7540, 2.5130), *p* = 0.064] (Fig. 1b).

### Diagnostic value of TEP lncRNA ROR in NPC

**Fig. 2:**
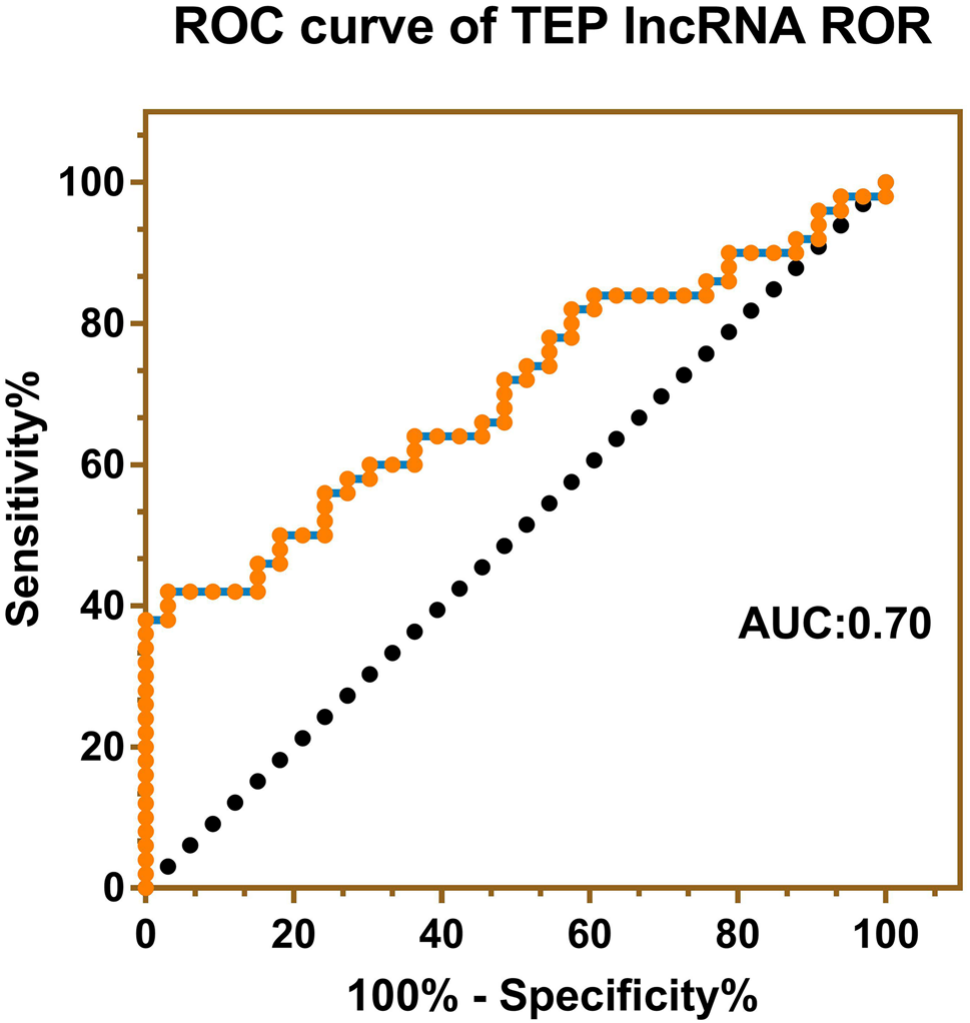
ROC curve of TEP lncRNA ROR for NPC

The area under the ROC curve (AUC) of TEP lncRNA ROR in diagnosing NPC was 0.70 (95% confidence interval: 0.59–0.81) (Fig. 2). And TEP lncRNA ROR < 0.0329 was set as the criterion in diagnosing NPC, with 0.30 of the Youden index. As a result, the sensitivity, specificity and accuracy for the diagnosis of NPC were 60, 70 and 63.9%, respectively.

### Expression level of TEP lncRNA ROR in different TNM stages

**Fig. 3:**
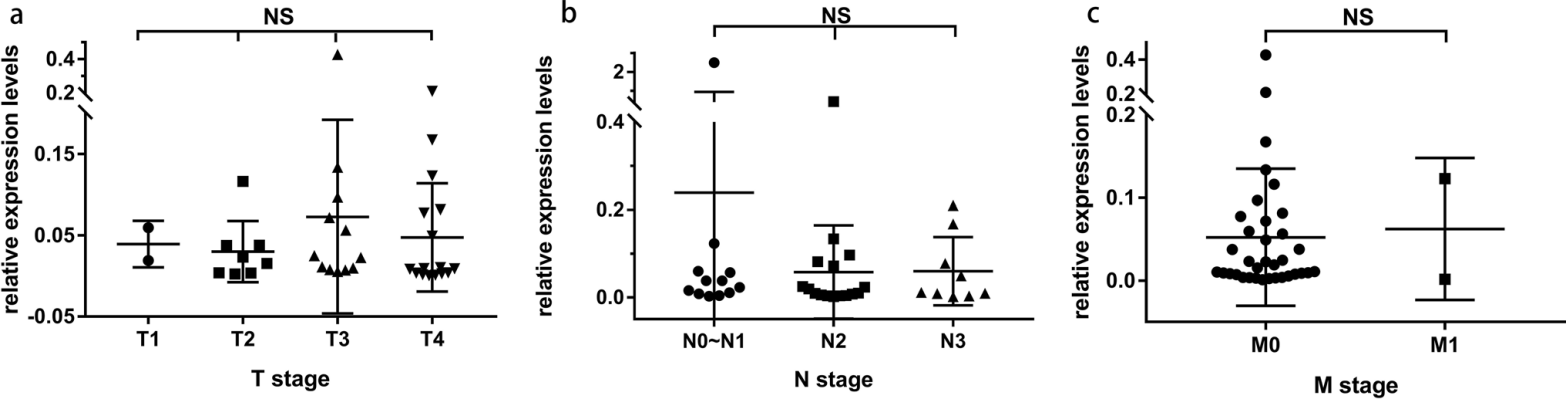
There was no significant difference in relative expression level of TEP lncRNA ROR among different TNM stages of NPC. **a** Relative expression level of TEP lncRNA ROR in T1–T4 stages, **b** Relative expression level of TEP lncRNA ROR in N0 ~ 1, N2, and N3 stages, **c** Relative expression level of TEP lncRNA ROR in M0 and M1 stages. Kruskal–Wallis test and one way ANOVA or Mann–Whitney *U* test was used for analysis

The expression level of TEP lncRNA ROR in NPC showed no significant difference among different T stages (*p* = 0.56) or N stages (*p* = 0.86) by Kruskal–Wallis test and one way ANOVA, respectively. In addition, no significant difference was found between M0 and M1 stages (*p* = 0.87) by Mann–Whitney *U* test (Fig. 3 and Table 2).

**Table 2:** Expression levels of TEP lncRNA ROR in different TNM stages

TNM stages	Median	25% percentile	75% percentile	*p* value
T stage				0.56*
T1	0.0393	0.0190	0.0596	
T2	0.0194	0.0036	0.0380	
T3	0.0239	0.0083	0.0906	
T4	0.0091	0.0031	0.0806	
N stage				0.86*
N0 ~ N1	0.0303	0.0085	0.0588	
N2	0.0144	0.0043	0.0792	
N3	0.0111	0.0057	0.1226	
M stage				0.87^a^
M0	0.0190	0.0073	0.0721	
M1	0.0623	0.0016	0.1230	

*Kruskal–Wallis test and one way ANOVA

^a^Mann-Whitney *U* test

### Correlations between TEP lncRNA ROR and clinical parameters of NPC subjects

We further investigated the relationship between TEP lncRNA ROR and NPC patients’ clinical parameters. NPC patients were divided into high-expression group and low expression group according to the cut-off point (0.0329) of TEP lncRNA ROR. The results showed that there were no significant correlations between TEP lncRNA ROR and age (*p* = 0.56), gender (*p* = 0.76), tumor classification (*p* = 0.83), node classification (*p* = 0.94) or metastasis classification (*p* = 0.78). However, low level of TEP lncRNA ROR correlated well with positive EBV DNA (kappa value = 0.314, *p* = 0.06). We further demonstrated that TEP lncRNA ROR and EBV DNA had similar diagnostic positive rate (58.3%) for NPC, and the combination of TEP lncRNA ROR and EBV DNA increased the positive rate to 74% (Table 3).

**Table 3:** Correlations between TEP lncRNA ROR and clinical parameters

Group	*N*	TEP lncRNA ROR			*p*
		Low (*n* = 30)	High (*n* = 20)	Low%	
Age	50	49.43 ± 2.056	47.6 ± 2.209	–	0.56^a^
Gender					0.76^b^
Male	33	19	14	57.6%	
Female	17	11	6	42.4%	
T					0.83^c^
T1	2	1	1	50%	
T2	8	5	3	62.5%	
T3	12	7	5	58.3%	
T4	16	10	6	62.5%	
N					0.94^c^
N0 ~ N1	12	6	6	50%	
N2	16	11	5	68.8%	
N3	9	5	5	55.6%	
M					0.78^c^
M0	35	21	14	60.0%	
M1	2	1	1	50.0%	
EBV DNA					0.99^d^
Positive	21	15	6	71.4%	
Negative	15	6	9	40.0%	
Positive rate of TEP lncRNA ROR: 58.3% vs. EBV DNA: 58.3%					Kappa: 0.314 *p*: 0.06^e^

^a^Independent *t* test

^b^Fisher’s exact test

^c^Chi-Square test for trend

^d^McNemar test

^e^Kappa test

## Discussion

In this study, we found that TEP lncRNA ROR was significantly lower in NPC patients than that in normal subjects, while no significant difference was found in plasma lncRNA ROR. ROC analysis showed that TEP lncRNA ROR had a sensitivity of 60%, specificity of 70%, and accuracy of 63.9% in diagnosing NPC, and the area under ROC curve was 0.70. Compared with EBV DNA, low level of TEP lncRNA ROR correlated well with positive EBV DNA, TEP lncRNA ROR and EBV DNA had similar diagnostic positive rate (58.3%) for NPC, and the combination of TEP lncRNA ROR and EBV DNA increased the positive rate to 74%.

For the function of lncRNA ROR in NPC, Li found that lncRNA ROR was significantly upregulated in NPC tissues and was highly associated with the proliferation, metastasis and apoptosis of NPC, in addition, the enrichment of lncRNA ROR played a critical role in chemoresistance through suppressing p53 signal pathway (Li et al. 2016); Hong found that the upregulation of lncRNA ROR could be inhibited by polyphyllin I, resulting in suppressing tumor growth and promoting apoptosis in vitro and in vivo (Hong et al. 2019). These two studies demonstrated that lncRNA ROR was overexpression in NPC tissues and played an important role in the progression of NPC. However, whether the expression level of lncRNA ROR in TEP is altered has not been studied yet. In our study, we found that TEP lncRNA ROR was significantly down-regulated in NPC patients as compared to normal subjects. We assumed that platelets might interact with NPC tissues indirectly via a vesicle-mediated communication or other pathway and then deliver lncRNA ROR to tumor tissues. As a result, TEP lncRNA ROR was down-regulated while upregulated in tumor tissues, promoting progression and metastasis of NPC.

A posterior rhinoscopy or endoscopy biopsy helps with the diagnose of NPC, however, the information acquired from a single biopsy is limited, and the invasiveness of this operation caused a limitation for repeated sampling. EBV detection is a useful marker in clinical practice which has been regarded as a biomarker for NPC in NCCN guidelines (Xue and He 2020). However, for those NPC patients without EBV infection, EBV detection is not available. Therefore, other useful biomarkers are urged for the diagnosis of NPC. The sensitivity of EBV DNA varies in different laboratories or regions, under the same conditions, we can use EBV DNA as a reference biomarker when evaluating diagnostic value for NPC. Our study showed that TEP lncRNA ROR correlated well with EBV DNA and had similar diagnostic performance to EBV DNA, the combination of TEP lncRNA ROR and EBV DNA increased the positive rate from 58.3 to 74%. These results indicated that TEP lncRNA ROR could be a good complement to EBV DNA, and as a non-invasive biomarker, TEP lncRNA ROR has great potential in clinical practice.

Considering the difficulty of obtaining tumor tissue in clinical practice, liquid-biopsy would be a better choice. Liquid biopsy is a minimally invasive, safe, and sensitive alternative or complementary approach for tissue biopsy and has shown great potential in cancer management (Bardelli and Pantel 2017). Although the tumor tissue biopsy is the current gold standard for cancer diagnosis, the information acquired from a single biopsy is limited and often fails to reflect the heterogeneity of the disease. Moreover, tissue biopsy is invasive which poses a limitation for repeated sampling. Liquid biopsy can therefore provide an accurate and comprehensive spatiotemporal snapshot of the tumor and its microenvironment on multiple levels (Sol and Wurdinger 2017). As one type of liquid-biopsy, TEP RNA detection provides a novel approach for cancer diagnosis. Our previous studies have found that TEP miR-34c-3p, miR-18a-5p, miR-18a-3p were upregulated in NPC and showed excellent diagnostic value (Sun et al. 2021; Wang et al. 2019). In this study, we further investigated that TEP lncRNA ROR was significantly down-regulated in NPC patients as compared to normal subjects. Interestingly, there was no significant difference about plasma lncRNA ROR expression. These results meant that platelets might interact with tumor cells and delivered their lncRNA ROR to tumor cells via vesicles or other ways. On the other hand, free lncRNA ROR in plasma could not be exchanged with tumor cells or was exchanged very little, therefore no significant difference was found in plasma. There are no relevant studies yet. Our results showed that detecting TEP lncRNA ROR was better than plasma when diagnosing NPC.

We determined quantity and quality of RNAs and guaranteed 1 ug of RNAs were used for reverse transcription. To make the results reliable, the H-actin RNA was set up as internal reference gene, and Ct value of H-actin RNAs were differ within 1. TEP lncRNA ROR has certain sensitivity, specificity and accuracy in the diagnosis of NPC, however, TEP lncRNA ROR shows no difference in each TNM stage and other clinical parameters, this may be due to the small sample size, and the results can only be preliminary at this stage. Moreover, a diagnostic consisting of more than a single lncRNA might strengthen the overall approach.

TEP lncRNA ROR as a liquid biopsy biomarker, is non-invasive and repeatable compared to tissue biopsy. What’s more, TEP lncRNA ROR could be a good complement to EBV DNA. In conclusion, TEP lncRNA ROR is a good biomarker for NPC diagnosis at current stage and more and more biomarkers will be found in the future through our research patten.

## Data Availability

The datasets generated during the current study are available from the corresponding author on reasonable request.

## Declarations

### Consents to participate

Informed consent was obtained from all individual participants included in the study.
